# Lighting the way: how the squid–*Vibrio* model can inform thermal impacts on symbiotic dynamics

**DOI:** 10.1242/jeb.251773

**Published:** 2026-04-27

**Authors:** Malcolm W. Thieme, Michele K. Nishiguchi

**Affiliations:** Department of Molecular and Cell Biology and Quantitative Systems Biology, University of California, Merced, Merced, CA 95343, USA

**Keywords:** Symbiosis, *Vibrio*, Thermal stress, Cephalopod, Mutualism, Bacteria

## Abstract

As global temperatures are shifting, so too is the landscape of organismal fitness and, by extension, the role of the symbiotic microbes they house. As these host–microbe partnerships grapple with changing environments, current research struggles to keep pace with the complexity of microbial symbioses acclimating, adapting and evolving as environmental conditions change around them. Wild-caught organisms have been used to test adaptation to extreme environments, but extrapolating and interpreting data on how separate partners within a symbiosis respond to detrimental conditions is difficult. The beneficial association between bobtail squids and bioluminescent *Vibrio* bacteria is a model that has been used for over three decades to uncover evolutionary and ecological mechanisms of symbiogenesis. The system is highly amenable to a broad range of physiological and molecular techniques and has been used to study many dimensions of symbiotic interactions. This beneficial association has demonstrated that host selection of environmentally available *Vibrio* symbionts can be influenced by various abiotic conditions, such as temperature. Complex biochemical communication has been charted extensively between host and symbiont, revealing universally conserved traits that are temperature sensitive. Additionally, temperature can influence co-evolution of the partners, and this system can be used to predict symbiotic cooperation over evolutionary time scales. While one model system cannot provide exhaustive insight, the bobtail squid–*Vibrio* mutualism has laid extensive, pioneering groundwork that can be used to develop targeted questions about symbioses under changing climates.

## Teaser

**Summary:** By using the bobtail squid–*Vibrio fischeri* model association, complex interactions within symbioses under thermal stress can be better understood.

## Introduction

**Fig. 1.**
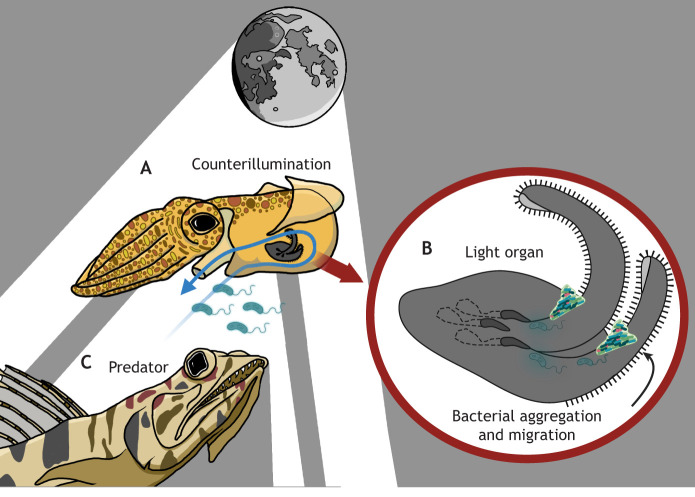
**Interactions between a squid host and its bioluminescent bacterial partner.** (A) The squid draws in seawater and environmental bacteria while respiring, which passes over the developing host light organ pores. (B) Bacteria form aggregates on the light organ appendages, where a bolus of mucus draws in planktonic cells that navigate into the organ through the pores and eventually colonize the crypt spaces and persist throughout the life history of the squid. (C) The ventral-facing light organ of the squid reduces the visibility of its silhouette, lessening the risk of predation. Predatory species, such as the lizardfish, cannot see prey silhouetted against the downwelling light in the water column, a behavior known as counterillumination. Created in BioRender by Thieme, M., 2026. https://BioRender.com/xqqp50d. This figure was sublicensed under CC-BY 4.0 terms.

Increasing environmental temperatures are impacting species' ranges and threatening local biodiversity, leading to concerns about the resilience of multiple ecosystems (Pigot et al., 2023; Pinsky et al., 2025). This question of environmental resilience increasingly acknowledges the essential role microbes play in responding to environmental perturbation at every ecosystemic level, including organismal fitness (Lennon et al., 2025; McFall-Ngai et al., 2013). Single-celled organisms have co-evolved with multicellular organisms since the inception of multicellularity and have asserted themselves as critical contributors to fitness in many diverse contexts (McFall-Ngai et al., 2013; Medina et al., 2022). As temperatures rise and organisms respond, a dimension of their response will be mediated by the microbes that they associate with. This layer of added complexity creates a challenge for researchers who must now interrogate the impacts of systemic climate change responses on highly interactive microbial populations for every organism. Thankfully, with the use of symbiotic model systems, larger sweeping trends can be unveiled elegantly through amenable proxies (Bosch et al., 2019). One such amenable proxy is the bobtail squid with its specialized light organ (see Glossary), where *Vibrio* bacteria are housed. Squids with these light organs are found in the families Loliginidae and Sepiolidae, the latter of which are known as bobtail squids (Nishiguchi et al., 2004). This interaction involves a squid host and its bioluminescent bacterial partner from the genus *Vibrio*, which provides an anti-predatory luminescence response known as counterillumination (Fig. 1) (Jones and Nishiguchi, 2004b). Extensively studied symbiotic interactions, like that between the bobtail squid and the bioluminescent bacterium *Vibrio fischeri*, provide a useful model to better understand complex host–microbe crosstalk (Soto et al., 2019; Nyholm and McFall-Ngai, 2021; Soto and Nishiguchi, 2021; Visick et al., 2021).

**Table:** 

**Glossary**
**Accessory nidamental gland**
A symbiotic organ found co-occurring with the light organ in female bobtail squids. This organ hosts a consortium of microbes which convey fitness to eggs laid by producing a protective jelly covering.
**Chaperone protein**
A protein which is robust to stressors that physically interacts with other proteins to keep them from denaturing. They are frequently expressed during times of stress; for example, when temperatures are outside a tolerable range.
**Ciliated**
The characteristic of having many small cilia. Cilia are the eukaryotic equivalent of bacterial pili and resemble microscopic hair-like structures on the surface of ciliated tissues.
**Holobiont**
A description of an organism and its associated microbial symbionts. This description is a synonym for ‘symbiotic system’ and can be used to describe any organism to emphasize the presence of complex cellular interactions taking place therein.
**Light organ**
A specialized organ that produces bioluminescent light. In bobtail squids, this organ is typically bilobed and is incorporated into the squid's ink sac. It hosts bioluminescent bacteria from which it derives light.
**Mantle**
The muscular structure encasing the visceral mass of a mollusk. In cephalopods, this structure is typically used for respiration and propulsion.
**Mutualism**
An interaction between two or more unlike organisms that conveys a fitness benefit to all parties associated.
**Psychrophilic**
Organisms, particularly bacteria, that preferentially live at colder temperatures. This typically describes a range of 15 to 20°C.
**Symbiont**
An organism living in long-term close association with another organism.
**Wrinkly biofilm phenotype**
A physical trait of certain bacteria that can form a specific biofilm structure containing unique biofilm-associated compounds. Wrinkly biofilms are known for being much more cohesive than their non-wrinkly morphotype, and are marked by distinctive visible invaginations, or wrinkles.

From a scientific perspective, luminescent symbioses are key examples for studying host–symbiont (see Glossary) interactions outside the framework of nutrient exchange, which has led to the rise of other similar model systems, including the cardinal fish–*Photobacterium* symbiosis (Douglas, 1995; Gould and Dunlap, 2019). Furthermore, an estimated 74% of marine life actively uses luminescence in some way: commercially relevant fish species such as brown trout, pink salmon, and Atlantic cod associate with luminescent bacteria, and swaths of the ocean as large as the state of Connecticut, USA, can become illuminated by milky overgrowths of luminescent bacteria (Nealson and Hastings, 2006; Fjellheim et al., 2007; Martini and Haddock, 2017; Bazhenov et al., 2019). This illustrates the prevalence of luminescent symbioses, and the existential risk a climate-driven shift in symbiotic cooperation could pose.

Luminescent symbionts, and symbioses at large, are impacted by novel environmental thermal stress. Having an amenable model such as the bobtail squid–*Vibrio* association enables improved understanding of complex aspects of how symbioses respond to thermal stress. Such responses can include the acclimation response of two partners simultaneously, potential range changes to one or both partners, evolutionary pressures on one or both partners, and potential changes to host–symbiont cooperation (Soto et al., 2012; 2014). These and other intricate dimensions of how an organism's fitness could be impacted by their symbionts responding to thermal stress are more easily disentangled using the model bobtail squid–*Vibrio* association (McFall-Ngai and Ruby, 1991; McFall-Ngai, 1999; Foster et al., 2002; McFall-Ngai, 2014; Belcaid et al., 2019; Bosch et al., 2019; Soto and Nishiguchi, 2021; Essock-Burns et al., 2023).

In this Review, we discuss how symbiont and organismal fitness might be impacted by novel temperatures. We focus on the bobtail squid–*Vibrio* symbiosis because it is a well-established research model. The squid–*Vibrio* symbiosis is amenable to many forms of experimentation and has revealed extensive data on both biotic and abiotic impacts on the development of the host–microbe relationship. First, we discuss how the symbiosis benefits the partners, then we focus on how thermal stress may alter the symbiosis under different contexts. This includes the impact of thermal stress on microbial recruitment into the host, how these stresses affect the free-living microbe and the range distribution of the host and microbe, and, finally, thermal stress over evolutionary time scales.

## How do luminescent bacteria contribute to host fitness?

### An introduction to the bobtail squid–*Vibrio* symbiosis

*Vibrio fischeri* bacteria provide their symbiotic host squid with bioluminescence for a behavior known as ‘counterillumination’ (Jones and Nishiguchi, 2004b; Stabb and Millikan, 2009) (Fig. 1). This behavior involves producing light to obscure the silhouette of the host animal from predators looking for prey which are backlit against light descending through the water column (Harper and Case, 1999). This is the leading hypothesis for why the squid has the highly specialized light organ, which is specifically optimized to accommodate light-producing bacteria. Bacteria are found free-living in the water column and are recruited into the light organ of juvenile bobtail squids, which are born without their light organ symbionts (Nyholm and McFall-Ngai, 2004; Soto et al., 2019). The juvenile bilobed light organ possesses three open pores and two mucus-covered, ciliated (see Glossary) appendages on each side (Nyholm et al., 2002; Nawroth et al., 2017; Kremer et al., 2018) (Fig. 1). As the juvenile squid respires, free-living *Vibrio* bacteria are taken up into the cavity of the squid's mantle (see Glossary) alongside circulating oxygenated seawater (Shadwick et al., 1990). During this time, 2.6 ml s^−1^ of seawater is ventilated past the juvenile light organ, exposing the unseeded symbiotic tissue to approximately 200–1500 *Vibrio* cells per second (McCann et al., 2003; Nyholm and Nishiguchi, 2008). These bacteria form an aggregate on the ciliated appendages and swim towards the three open pores of the light organ (Yip et al., 2006; Schwartzman and Ruby, 2016a; Visick et al., 2021). Once in the pores, the squid host exposes the bacteria to reactive oxygen species such as nitrous oxide, whilst enticing *Vibrio* into the light organ crypts with chitin-derived chemical signals (Davidson et al., 2004; Kremer et al., 2013; Nyholm and McFall-Ngai, 2021). These measures ensure that only symbiotic *Vibrio* bacteria, and almost exclusively *V. fischeri*, are capable of passing from the environment into the deep symbiont-housing crypts of the squid light organ to colonize the squid. It takes as few as five *Vibrio* bacterium cells to initiate this symbiosis cascade (McCann et al., 2003; Kremer et al., 2013).

The symbiotic light organ contains a lens and reflector proteins and can modulate light output by flexing an aperture through its opaque ink sac. This complex machinery does not develop unless the squid is exposed to bacteria capable of producing luminescence (Visick et al., 2000; Nishiguchi et al., 2004; Koch et al., 2013; Kremer et al., 2018). The partnership operates in two directions: the bacteria receive nutrients and are dispersed and protected from potentially deadly phages (Wier et al., 2010; Lynch et al., 2022), while the squid benefits from the luminescent output that it cannot produce itself.

### How might adaptation of the symbiosis to thermal stress impact host fitness?

The novel luminescence function conferred to the squid by *Vibrio* bacteria is of particular interest when studying symbioses under environmental stress. As discussed in Voolstra and Ziegler (2020), symbioses may possess the ability to buffer thermal stress via adaptation, yet it can be difficult for many symbiotic bacteria to adapt to novel stressors, as they often have highly reduced genomes (Wernegreen, 2012). This can be particularly challenging for symbiotic associations under changing environmental conditions, when the host depends on the symbiont for novel fitness contributions. If *V. fischeri* loses the ability to luminesce, they will be rejected by the host squid, which will be susceptible to predation with no means of counterillumination on its own (Castle, 2013).

The potential for luminescence to be lost under thermal stress is a real possibility. While heat-adapted bacteria can increase their luminescent output under non-stressful conditions (Cohen et al., 2019), luciferase – the protein responsible for light production – denatures quickly under higher temperatures (Lohrasbi-Nejad et al., 2016). The impact of this denaturing behavior is highly variable across bacterial species, but *V. fischeri* have shown reduced luminescence at 31°C (Williams et al., 2019; Calogero et al., 2022; Deeva et al., 2022). This is a temperature observed in the northern Great Barrier Reef and that overlaps with the known range of the bobtail squid–*Vibrio* association (Huang et al., 2024). Additionally, warming waters are known to reduce environmentally available oxygen, owing to both the solution losing some capacity to retain dissolved gas and an increase in biological oxygen demand as decomposition becomes more prevalent (Yavuz, 2025). The light-producing luciferase reaction is dependent on oxygen, so heat could have an additional impact on luminescent fitness (Adams and Miller, 2020). *In vitro* trials have shown that oxygen concentrations as high as 5% in solution reduce the output of the luminescence response (Moriyama et al., 2008). A reduction in luminescent fitness in response to direct thermal stress, or a secondary consequence of thermal stress, has the potential to impact the many species that form luminescent symbioses, including the bobtail squid–*Vibrio* bacteria association.

There may be additional benefits that host organisms glean from their symbiotic partners which are less obvious. *Vibrio fischeri* digest reactive oxygen species readily, which is not only important for their colonization fitness but also hypothesized to be intertwined with the evolutionary origin of bacterial bioluminescence (Szpilewska et al., 2003; Davidson et al., 2004; Łyżeń and Węgrzyn, 2005; Miyashiro and Ruby, 2012). In multiple experiments, the impact of thermal stress on clutches of cephalopod eggs has led to an increase in reactive oxygen species associated with a decrease in hatching success (Rosa et al., 2012; Kuan et al., 2022; Otjacques et al., 2025b). The mediation of reactive oxygen species might also be a secondary host fitness contribution from symbioses.

The fitness advantages that symbiotic bacteria impart onto their host organisms are diverse and not always well understood. The anemone *Nematostella vectensis* hosts a symbiont, *Tenacibaculum*, which is a bacterium hypothesized to buffer the impact of UV radiation stress for the host (Starcevic et al., 2008). The bivalve *Thyasira gouldi* is known to take up *Endoriftia persephone* symbionts and assimilate magnetic ions into their cells for the purpose of enticing other symbionts to orient towards the host (Dufour et al., 2014). Even bobtail squids with their familiar *V. fischeri* symbionts have a secondary, more complex, symbiotic organ called the accessory nidamental gland (see Glossary), which deposits a bacteria-rich jelly onto their eggs to dissuade overgrowth by harmful marine microbes (Kerwin and Nyholm, 2017; McAnulty et al., 2023; Vijayan et al., 2024). The interaction of host and symbiont is complicated and there are multiple dimensions through which a symbiont can supplement fitness in their host. By using a symbiotic system to study how thermal perturbation affects both host and partner fitness, the nuanced impacts of environmental change on symbioses can be illuminated.

## Is symbiotic recruitment impacted under thermal stress?

The recruitment of the symbiont is critical for many symbioses, with notable examples including the zebrafish gut, vestimentiferan tubeworm coelomic cavity and human vaginal microbiome community (Cavanaugh et al., 1981; Gilbert, 2014; Hill et al., 2022). These complex relationships between the host and multiple symbiotic partners make trends difficult to decipher through the static of a noisy microbial consortium. The comparatively simple nature of *Vibrio* recruitment in squid has been studied extensively, revealing multiple levels of molecular cross-talk between the partners (Kremer et al., 2013; McFall-Ngai, 2014). In this way, the bobtail squid–*Vibrio* system has provided insight into the impact of rising temperatures on bacterial recruitment (Jones et al., 2006; Soto et al., 2008; Schwartzman and Ruby, 2016a; Mandel and Dunn, 2016; Cohen et al., 2019; Soto and Nishiguchi, 2021; Visick et al., 2021; Pipes and Nishiguchi, 2022).

### Bobtail squid–*Vibrio* molecular crosstalk

**Fig. 2.**
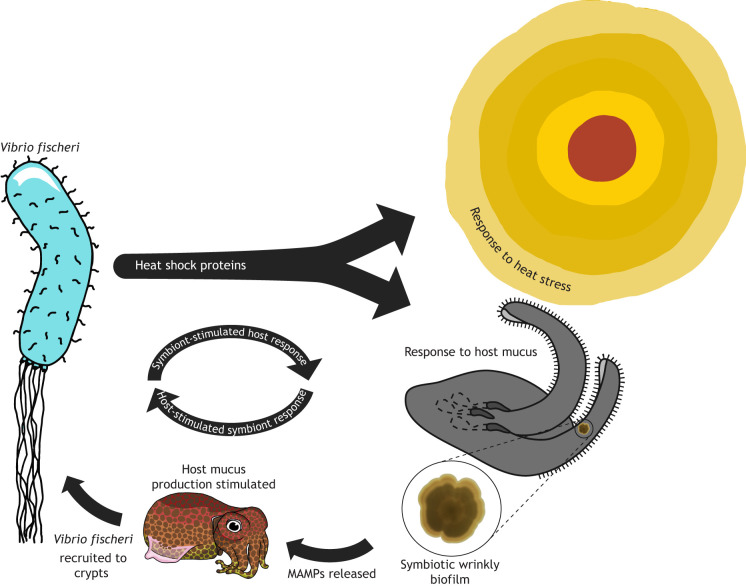
**Molecular crosstalk between squid and *Vibrio*.** Bacteria, in response to heat stress, produce heat shock stress proteins. These heat shock proteins have been shown to have a direct link to development of the symbiotic wrinkly biofilm, a phenotype the bacteria adopt following exposure to host mucus on the ciliated appendages of the light organ. These symbiotic biofilms allow for the production and transfer of microbe-associated molecular patterns (MAMPs), which stimulate responses in the host organism. One such response includes changes in the regulation of mucus production, which further incentivizes *V. fischeri* recruitment. This illustrates how the thermal stress response, and complex host–symbiont molecular communication, directly share key molecular components. Created in BioRender by Thieme, M., 2026. https://BioRender.com/yvwjo5x. This figure was sublicensed under CC-BY 4.0 terms.

Once taken up into the squid's mantle, the complex communication network between the bobtail squid and their establishing *Vibrio* symbionts commences (Essock-Burns et al., 2021). *Vibrio* bacteria contact the mucus-coated ciliated appendages of the juvenile light organ, and an aggregation factor called RscS is upregulated, which stimulates gene expression to produce a wrinkly biofilm phenotype (see Glossary) (Fig. 2) (Yip et al., 2006). The wrinkly biofilm, which is controlled by the *syp* regulon, is important for *V. fischeri*’s strong symbiotic behavior (Yip et al., 2005; Morris and Visick, 2013; Soto et al., 2019; Fung et al., 2024). In the wrinkly biofilm state, *V. fischeri* produces outer membrane vesicles (OMVs) which contain critical microbe-associated molecular patterns (MAMPs), including peptidoglycans, which induce physical changes in the symbiotic light organ of the squid (Koropatnick, 2004; Shibata and Visick, 2011; Aschtgen et al., 2015; Moriano-Gutierrez et al., 2020). Tissues of the squid possess peptidoglycan receptor proteins (PGRPs), which are highly conserved across eukaryotes and trigger a conserved MAMP response pathway driven by NF-κB signaling (Goodson et al., 2005; Troll et al., 2009, 2010; Olaso et al., 2022). The light organ subsequently undergoes a slew of microbe-mediated morphological changes due to these bacterial signals, which include changes in mucus secretion that continue to promote *V. fischeri* recruitment (Nyholm et al., 2002; Schwartzman and Ruby, 2016a; Kremer et al., 2018). The symbiosis is highly specific, yet environmental conditions outside the host can affect the symbiosis in multiple facets (Soto and Nishiguchi, 2021; Soto et al., 2019). In this way, the symbiont and host respond to and induce changes in each other.

### How might temperature impact host–symbiont crosstalk during recruitment?

Environmental thermal stress has major effects on host–symbiont communication. The bacterial aggregation factor RscS allows specific *Vibrio* species to congregate near the light organ pores, while non-symbiotic bacteria are outcompeted by symbiotic *Vibrio* (Nyholm and McFall-Ngai, 2003). RscS is also needed for the symbiotically critical wrinkly biofilm behavior, driven by the *syp* regulon, so if *rscS* or *syp* is mutated or inhibited, *Vibrio* are unable to colonize effectively (Yip et al., 2005, 2006). Interestingly, both RscS and *syp* depend on chaperone proteins (see Glossary) to function (Fig. 2) (Shibata and Visick, 2011; Brooks et al., 2014; Brooks and Mandel, 2016). These chaperones are DegP (for RscS) and DnaK/DnaJ (for *syp*), which are highly conserved heat shock proteins found in many bacteria (Spiess et al., 1999; van der Veen and Abee, 2010). In the absence of these heat shock proteins, colonization deficiencies occur which mimic the behavior of bacteria with mutated RscS and *syp* regulation (Shibata and Visick, 2011; Brooks et al., 2014; Brooks and Mandel, 2016). The presence of heat shock proteins integrated into the biochemical processes needed to facilitate symbiotic initiation (Fig. 2) could indicate that bacterial symbiotic behavior possesses a degree of thermal robustness. This does not appear to be the case, however, as symbiosis-associated phenotypes are altered under *in vitro* thermal stressors, and higher concentrations of symbiont are needed to colonize host squid under increased temperatures (Soto et al., 2008; Williams et al., 2019; Otjacques et al., 2025a). These heat stress chaperones seem to play a critical role in symbiosis function, but there is no indication that their role in symbiosis is buffering thermal stress. Additionally, research in maternally transmitted symbiotic systems (as opposed to environmentally transmitted) has shown that genome reduction is common in symbiotic bacteria, preserving only the bacterial genes needed for basic survival, including heat shock proteins (McCutcheon and Moran, 2011). Such molecular mechanisms are needed for bacterial thermal tolerance and are intrinsically linked to symbiotic formation and maintenance, but do not appear to be making these symbiotic relationships robust to thermal changes.

Molecular communication mechanisms of bobtail squid hosts also show sensitivity to thermal stress. Squid PGRPs are needed to detect and respond to *Vibrio* bacteria, and PGRPs are highly conserved proteins found in all eukaryotes (Werner et al., 2000; Dziarski, 2004; Steiner, 2004; Coteur et al., 2007; Troll et al., 2009, 2010). Previous studies of symbiotic behavior in pest species *Aedes aegypti* (yellow fever mosquito) and *Ostrinia furnacalis* (Asian corn borer) demonstrate changes in PGRP regulation under heat stress, which correlates with shifts in microbiome composition (Chen et al., 2019; Ferreira et al., 2025 preprint). Additionally, PGRPs in bobtail squids trigger the Toll/NF-κB response pathway (Goodson et al., 2005). This is a highly conserved biochemical pathway with homologs found in most plant and animal species (Hoffmann, 1999; Asai et al., 2002; Nürnberger and Brunner, 2002). In bobtail squids, thermal stress experiments have shown a decrease in *Vibrio* colonization occurring alongside a downregulation of NF-κB expression (Otjacques et al., 2025a,b). This evidence suggests a direct thermal impedance to squid host regulation of symbiont recruitment.

Selection for specific microbial symbionts by the host is a key behavior seen in other symbioses outside the *Vibrio*–squid system, including in *Hydra* microbiomes, rainbow trout hindgut communities and even human gut microbiomes (Gilbert, 2014; Maritan et al., 2024). It is common for symbiotic bacteria aggregation to depend on heat shock proteins to function (Ram et al., 2005; van der Veen and Abee, 2010). Animal PGRPs and the NF-κB pathway are conserved structures with applications in bacterial interaction and response, and their transcription is correlated with thermal stress (Hoffmann, 1999; Werner et al., 2000; Asai et al., 2002; Nürnberger and Brunner, 2002; Dziarski, 2004; Steiner, 2004; Coteur et al., 2007; Chen et al., 2019; Otjacques et al., 2025b; Ferreira et al., 2025 preprint). Given these parallels, the bobtail squid–*Vibrio* symbiosis can offer critical insight into the resilience of symbiont recruitment under thermal stress.

## How are symbiotic bacteria species responding to thermal stressors?

In response to stressful conditions, such as those imposed by extreme temperatures, animals must choose to move, adapt or die (Parmesan, 2006; Urban, 2015, 2024; Pinsky et al., 2025). However, animals are more than just eukaryotic cells: they are a complex consortium of symbioses (McFall-Ngai et al., 2013). For a single bacterium, the challenge of stressful conditions is difficult to overcome via behavioral change, like migration. Instead, bacteria are usually either resigned to death or required to undergo a rapid alteration in gene expression in the face of a stressful environment (Henkin et al., 2020). This extreme alteration of microbial genetic regulation can fundamentally change the life history, metabolic processes and even reciprocal symbiotic function of the bacteria (Soto et al., 2008; Schwartzman and Ruby, 2016b; Carvalho et al., 2025). The bobtail squid–*Vibrio* symbiosis can offer a unique perspective on the symbiotic function of a bacterium, as *Vibrio* bacteria are a diverse class of symbionts – comprising free-living, mutualistic and pathogenic species – and the delineation between host assistance and host sabotage is often determined by environmental conditions.

*Vibrio fischeri*’s symbiotic relationship with bobtail squid is a glowing example of a mutualism (see Glossary), but despite its reputation, *V. fischeri* retains behavior and function analogous to that of other *Vibrio* pathogens. Evolution does not appear to cluster pathogenic and mutualistic *Vibrio* traits separately, as suggested by the lack of monophyletic clustering of *Vibrio* 16S rDNA sequences between mutualists and pathogens (Nishiguchi and Nair, 2003). Additionally, whole-genome sequencing has uncovered pathogen-associated capabilities in *V. fischeri* – including genes shared with other pathogens, such as those encoding bacterial RTX toxin and toxin-delivering secretion machinery (Whistler and Ruby, 2003; Ruby et al., 2005; Gonzalez-Moreno and Nishiguchi, 2025). *Vibrio fischeri* also has many genes with homologs that are associated with toxin production and the stress response in *Vibrio cholerae*, including ToxR, ToxS, HtpG and RpoH (Reich and Schoolnik, 1994; Mandel et al., 2008). These are still found in *V. fischeri* despite a 70-million-year mutualistic association with bobtail squids (Sanchez et al., 2021). The homologies with pathogenic pathways make it less surprising that *V. fischeri*, despite being an ally to bobtail squids with no evidence of pathogenesis in nature, imposes a lethal pathogenic threat to brine shrimp, zebrafish embryos and a swathe of other fish and shrimp species (Gaddy et al., 2025; López et al., 2017). Even literature on *V. fischeri* lab cultivation cautions that it is totally non-pathogenic, as long as typical growth conditions are maintained (Christensen and Visick, 2020). For an animal host, the delineation between beneficial partner and deadly infection, even for renowned mutualists, can be context dependent.

There is an abundance of examples where the context-dependent factor determining symbiont cooperation or symbiont parasitism is temperature, especially for *Vibrio* bacteria. *Vibrio salmonicida* is an opportunistic pathogen responsible for ‘cold water disease’, where water temperatures below 12°C impact the expression of the master quorum sensing regulator LitR that causes changes in the expression of *syp* biofilm production, which is the same biofilm regulator needed for host association between the bobtail squid and *V. fischeri* (Sanches-Fernandes et al., 2022; Hansen et al., 2014). This temperature-sensitive shift in biofilm regulation allows the bacterium to invade fish hosts, spelling death for trout, cod and salmon (Khider et al., 2018). Warming waters also cause shifts in *Vibrio* pathogenicity. *Vibrio coralliilyticus* is a broad-ranging marine pathogen which infects unicellular algae, trout, shrimp, oysters and many other hosts, but it is most notable for causing the ‘black band disease’ devastating coral reefs (Kimes et al., 2012; Hmelo, 2017). This pathogen is dormant at 24°C, living in and not harming the coral, but becomes deadly at 27°C (Gibbin et al., 2019; Kimes et al., 2012). The same influence of heat on pathogenesis is also observed in *Vibrio parahaemolyticus*, a conditionally pathogenic *Vibrio* seen harmlessly associating with many host species, including shellfish consumed by humans and bobtail squid such as *Euprymna scolopes* (Nyholm et al., 2000; Urmersbach et al., 2015). However, at higher growth temperatures, *V. parahaemolyticus* increases expression of DnaK, a bacterial heat shock response protein, causing a subsequent increase in biofilm formation. This leads to *Vibrio* overgrowth in the host, making them pathogenic to both the host and any humans unfortunate enough to consume infected hosts as seafood (Sanches-Fernandes et al., 2022; Urmersbach et al., 2015). In fact, changes in sea surface temperature have been shown to have a direct correlation to increases in multiple *Vibrio* pathogen outbreaks in humans (Patz et al., 2005; Baker-Austin et al., 2013). Subtle shifts in average temperature have the potential to turn the unassuming microbial landscape, which animals interact with constantly, into a disease-filled minefield.

A stark reminder of this possibility occurred in 2013. A heatwave led to an outbreak of ‘Sea Star Wasting Disease’ (SSWD), which nearly killed off important marine keystone species (Hewson et al., 2024). This drastically impacted the ecology of the American Pacific Northwest intertidal and subtidal zones. While the exact cause of this environmental catastrophe is still poorly understood, it is widely speculated that the change in environmental temperature caused a breakdown in host–microbe interactions for sea stars (Lloyd and Pespeni, 2018). Recently, over a decade after the initial devastation, a leading candidate cause for the infection has been identified as *Vibrio pectenicida* (Prentice et al., 2025). These complex, temperature-sensitive interactions can be better understood through a model system which may help provide early insight.

The bobtail squid–*Vibrio* system possesses extensive utility in understanding host–symbiont dynamics, especially in pathogenic contexts. The metabolic flexibility of *V. fischeri*, its similarity to other pathogens and the parallels between symbiotic *Vibrio–*squid light organ association dynamics and pathogenic *Vibrio* association dynamics with human gut tissue make it an effective and safe analog for understanding pathogen behavior (Nishiguchi et al., 2008; Dunn, 2012). As microbes respond to changing temperatures, animal hosts will be subject to the fallout from the microbial responses, which is where model systems such as the bobtail squid–*Vibrio* symbiosis can help provide insight into how symbiotic microbes acclimate.

## How is temperature impacting the global distribution of symbiotic bacteria?

Abiotic factors such as temperature have been shown to impact the global distribution of microbes, with changing latitudes influencing variation in local microbial richness (Thompson et al., 2017). Temperature, in many contexts, directly impacts the composition of animal gut microbiomes (Sepulveda and Moeller, 2020). Ectotherms, in particular, are sensitive to increases in temperature that lead to digestive deficiencies and altered gut microbial diversity, and compromise a host's ability to exclude pathogenic taxa (Fontaine et al., 2018). These environmentally induced shifts in microbial communities are of interest when studying organismal health but understanding the complexity of shifting microbiomes has generated a need for model systems, such as the squid–*Vibrio* relationship.

A common misconception regarding the bobtail squid–*Vibrio* association is that the relationship, which can be binary under sterile lab conditions, remains binary in the wild. Sepiolid and loliginid squids will often have multiple bacterial strains occupying a light organ at once, and multiple species of bacteria are capable of forming symbioses, including *Vibrio logei* and *Vibrio harveyi* (Fidopiastis et al., 1998; Nishiguchi et al., 1998; Guerrero-Ferreira et al., 2013; Pérez-Ferrer et al., 2024). This slight propensity for microbial diversity allows for basic questions of microbiome shift to be broached in the comparatively simple squid light organ model.

The impact of abiotic factors on the distribution of microbes also affects the squid's microbial symbionts. Environmental samples from waters known to be inhabited by bobtail squids demonstrate fluctuations in the presence and concentration of luminescent symbionts (Lee and Ruby, 1994; Jones et al., 2007). Earlier studies measured higher concentrations of symbionts near populations of hosts in Hawaii, but exact concentrations of bacteria in the seawater were highly subject to tides and currents (Lee and Ruby, 1994). A more geographically extensive study of water samples showed that differences in the concentration of symbionts found in water samples from France, Hawaii and Australia were most directly impacted by seasonality (Jones et al., 2007). These findings seem to reflect studies of the composition of microbial communities discovered in the light organs of squids from those areas (Jones et al., 2006; Zamborsky and Nishiguchi, 2011; Coryell et al., 2018; Pérez-Ferrer et al., 2024). Nested clade analyses of host squids collected from Hawaii, France, Australia and the Philippines show variable patterns of symbiont and host distribution, which is attributed to abiotic factors, including temperature and currents, that dictate the range of *Vibrio* symbionts within and across seasons (Jones et al., 2006; Zamborsky and Nishiguchi, 2011; Coryell et al., 2018; Pérez-Ferrer et al., 2024). Additionally, another microbial organ in bobtail squids, the accessory nidamental gland, impacts squid health and is also sensitive to environmental microbe composition (McAnulty et al., 2023). Taken together, this demonstrates that abiotic factors influence the environmental distribution of symbionts, consequently impacting which symbiotic species the squid ultimately associates with.

These environmental studies are entrenched in a battery of potentially impactful variables, but when isolated in the lab, the bobtail squid–*Vibrio* symbiosis is still impacted by changes in temperature. Previous work revealed a direct correlation between temperature and symbiont selection (Nishiguchi, 2000; Otjacques et al., 2026). *Vibrio logei* outcompetes *V. fischeri* in dual infection experiments at 18°C, with a ratio ranging from 80% to 90% *V. logei* to a mere 10% to 20% *V. fischeri*, and the inverse competition ratios occur at 26°C (Nishiguchi, 2000). This same preference for the psychrophilic (see Glossary) luminescent bacterium *V. logei* at colder temperatures is observed in fish across the Bering and Okhotsk Seas, with their gut communities boasting 89.5% *V. logei* to 10.5% *Photobacterium phosphoreum* in the winter, and an inverse 12.1% *V. logei* to 87.9% *P. phosphoreum* in the summer (Bazhenov et al., 2019). Warmer temperatures of 30°C make it challenging for bobtail squid to form associations with *V. fischeri*, with more bacteria being required to form a successful symbiosis (Otjacques et al., 2025a). Of note is the breakdown of *V. fischeri* luminescence function at 31–32°C, which is a trait critical to the formation of the symbiosis (Williams et al., 2019; Visick et al., 2000). If the squid host can adapt to the stressors of higher environmental temperatures, it may prefer a different symbiont which can luminesce more proficiently under higher temperatures. Previously, *V. harveyi* was described as a potential squid symbiont or pathogen (Guerrero-Ferreira et al., 2013). *Vibrio harveyi* also has a luminescent response that is tolerant to higher thermal ranges, peaking in function at 35°C (Deeva et al., 2022). Just as there is a temperature-mediated discrepancy for squid symbiont preference between *V. logei* and *V. fischeri*, a shift in preference may occur between *V. fischeri* and *V. harveyi* as environmental temperatures warm.

Shifting environmental conditions play a key role in microbiome composition and allow a window into understanding how broad trends in microbiome flexibility are affected. This is particularly true in many animal groups, including cephalopods (Nishiguchi, 2000; McAnulty et al., 2023; Bennice et al., 2024). The bobtail squid–*Vibrio* system enables research that addresses whether one strain or species of luminescent symbiont is more preferable to the host than another under higher temperatures and whether there are changes in the distribution of *Vibrio* symbionts in hosts and host-rich waters over 5, 10 or 20 years as local climates shift. As a case study, *Pomacea canaliculata* is a highly invasive freshwater snail currently plaguing Asia, Europe and North America. While sensitive to thermal stress, its microbiome has shown a remarkable ability to adapt, becoming more diverse after only 28 days under temperatures as high as 35°C (Li et al., 2022). Understanding how a symbiosis may break down or adapt to thermal stress is an important dimension of safeguarding fragile animals and ecosystems. This is where using a symbiotic model system can take a complex ecological problem and condense a single case study down to the fundamentals of host fitness, inter-organismal molecular interaction and symbiont availability.

## Are symbiotic bacteria adapting to changes in temperature over evolutionary time scales?

Another key topic dictating host fitness is the co-evolution of both partners. More specifically, the extensive degree to which co-evolution with microbial partners shapes an animal's evolutionary trajectory (McFall-Ngai et al., 2013; Medina et al., 2022). The bobtail squid–*Vibrio fischeri* mutualism can also be used as a case study for co-evolving partners (Nishiguchi et al., 1998). Despite *V. fischeri* persisting quite effectively in a range of free-living niches, an evolutionary influence on both host and symbiont is evident within their speculated 70-million-year-old relationship, despite the relationship's seemingly non-mandatory nature (Nishiguchi et al., 1998, 2004; Jones and Nishiguchi, 2004a; Sanchez et al., 2021). For squid, shallow genome sequencing and comparative anatomy allude to a monophyletic origin of the symbiotic light organ (Nishiguchi et al., 2004). Additionally, luminescent output is highly variable across *V. fischeri* strains, which is thought to be the result of rapid evolution due to the pressures (including environmental temperature) of either free-living or host-associated life history strategies (Bose et al., 2011). The evidence of this co-evolutionary relationship is further supported by host preference for strains of bacteria native to specific regions. *Vibrio fischeri* strains isolated from both Hawaiian and Japanese *Euprymna* squid species can be introduced into Hawaiian *E. scolopes* simultaneously, which colonize the light organ with a ratio 98:2 native versus non-native. Interestingly, colonization between two non-native *V. fischeri* strains (Hawaiian versus Japanese) in Australian *Euprymna tasmanica* yields a ratio of 3:97 in favor of the Japanese *Vibrio* strain (Nishiguchi et al., 1998; Nishiguchi, 2002). The *V. fischeri* strain in these studies was isolated from the Japanese squid *Euprymna morsei* and is classified as a dominating strain, but it is unable to dominate the native strain of Hawaiian bacteria in its native host animal even though it can outcompete other strains in the Australian squid (Bongrand et al., 2020). This may be due to the differences in tropical and temperate climates where the Hawaiian and Australian squids were isolated from, such that the Australian climate is more reminiscent of the climate where the Japanese bacteria were isolated. These patterns of host preference are found across all squids in the *Euprymna* genus and their associated *V. fischeri*, and these patterns of colonization proficiency independently align with their hypothesized evolutionary development (Nishiguchi and Nair, 2003; Bongard et al., 2020). Understanding a species' evolutionary history makes it possible to speculate about the impacts of abiotic factors on the future of symbiotic relationships, but the bobtail squid–*Vibrio* system offers the additional advantage of manipulating the system *in vitro* for further study.

One method widely used to test how evolutionary pressures impact strain specificity and competitive dominance is experimental evolution. Since 1880, this experimental method has been used extensively to study organisms with short generation times (Toal, 2013). Experimental evolution entails maintaining a series of replicate populations of genetically identical organisms under pre-set test conditions for many successive generations to see what – if any – influences the test conditions have on the experimental populations (Travisano et al., 1995). While bobtail squids have a lifespan and genetic complexity that makes such experimentation on the host challenging, *V. fischeri*, as a bacterium with a generation time measurable in minutes, is an ideal test subject for experimental evolution studies (Soto et al., 2012; Soto and Nishiguchi, 2014). By experimenting on the faster-adapting partner in the symbiosis, influences of environmental conditions on the relationship between host and symbiont can be elucidated.

Shorter series of experimental evolution work on the system have demonstrated that bacteria cultured outside the squid for multiple successive generations become more proficient colonizers, despite there being no host to interact with (Soto et al., 2019). Conversely, long-term studies revealed that non-native *V. fischeri* symbionts isolated from different squids, fishes and seawater demonstrate changes in luminescent output and carbon source utilization after being serially passaged through a non-native squid host for 300 to 500 bacterial generations (Schuster et al., 2010; Soto et al., 2012, 2014). This verified a level of symbiont malleability which tailors the *Vibrio* bacteria towards becoming a more favorable partner to their host over the course of many successive generations. Outside the host, abiotic factors such as pH can influence the growth and fitness of *V. fischeri* symbionts over many successive generations (Cohen et al., 2020; Nourabadi and Nishiguchi, 2021). After 2000 generations of adaptation under a more acidic pH of 6.0–6.5, host colonization increased by as much as 50% compared with the ancestor, and bacterial luminescence nearly doubled relative to the ancestor, over the course of only 600 bacterial generations (Nourabadi and Nishiguchi, 2021).

In addition to pH, the influence of temperature also imposes a known evolutionary pressure on bacteria, including *V. fischeri* (Nyholm and Nishiguchi, 2008; Soto et al., 2008). *Vibrio fischeri* strain ET00-7-1 isolated from the Australian squid *E. tasmanica* was selected under five different thermal test conditions: 8, 21, 28, 34 and 8/34°C fluctuating, maintained for 2000 bacterial generations (Cohen et al., 2019). Squid infected with the 34°C- and fluctuating temperature-adapted bacteria had their light organs colonized at concentrations of 1×10^6^ to 1×10^9^ colony forming units (CFU) ml^−1^ and the infection inoculum for these test groups produced a luminescent output of 1×10^70^ to 1×10^80^ relative luminescence units (RLU) CFU^−1^. These values far exceed the output of the un-adapted ancestor ET00-7-1, which infects the squid at concentrations of 1×10^4^ CFU ml^−1^ and luminesces at a relatively dim 1×10^20^ RLU CFU^−1^. Much like more acidic conditions, warmer temperatures promote *V. fischeri* symbiotic competence, but so far this has only been tested for one strain of *V. fischeri* isolated from Australia, which has annual thermal fluctuations reminiscent of a temperate climate (Anderson et al., 2005; Cohen et al., 2019). Temperate bacteria tend to be more robust to changing environmental conditions, but the ability of a tropical *V. fischeri* symbiont to adapt and convey fitness benefits is still being investigated in the context of its beneficial association with squid (Hochachka and Somero, 2002).

The impact of temperature on bacterial evolution, especially across diverse ecological contexts, is a key driver of symbiosis initiation and can directly affect bacterial generation time (Soto and Nishiguchi, 2021; Sheikh et al., 2022). Experimental evolution methods have been adopted in other systems to disentangle how abiotic factors, such as temperature, impact the overall health of the partnership. These experiments have inspired artificial transplant work to study the impacts of adapted microbiomes on organismal health. In *Drosophila melanogaster*, transplanting the microbiome of flies artificially adapted to 31°C increases the upper thermal limit that a naive fly can physically tolerate (Moghadam et al., 2017). Similar work conducted in the anemone *Nematostella vectensis* confirmed that the microbiomes derived from a 2 year thermal acclimation experiment could be transplanted to new hosts, increasing their survival rate under heat stress by 20% (Baldassarre et al., 2022). Understanding this phenomenon of rapid organismal adaptation to thermal stress through microbiome modification is especially attractive in coral research, given that corals are important keystone species, are characteristically slow growing and are highly dependent on symbioses (Peixoto et al., 2017). The hard coral *Acropora tenuis* was introduced to two thermally adapted symbionts and demonstrated that one symbiont (*Cladocopium proliferum*, strains SS1 and SS8) conveyed bleaching tolerance to the coral under simulated heat stress of 31°C, while corals with wild-type symbionts struggled (Quigley et al., 2023). One current hypothesis is that the microbiome of an organism can adapt to a novel stress, such as an altered temperature range, within the span of an organism's lifetime, providing a level of evolutionary adaptation within one generation of the host organism (Voolstra and Ziegler, 2020). This is further supported by the expanding field of microbiome experimental adaptation work; including the experimental evolution work in germ-free zebrafish, which has shown that symbionts can become significantly more proficient at associating with the host after only 80 days of live passaging through the zebrafish (Robinson et al., 2021).

Temperature stress is a fascinating topic when studying the evolution and real-time adaptation of symbioses, as it is thought to be a key initial driver for many instances of symbiotic development (Kokou et al., 2018). Studying how a fast-adapting partner handles novel stressful conditions provides insight into the stability and persistence of a holobiont (see Glossary) in a changing world and how such relationships can buffer the impacts of thermal stress on organisms. For complex and time-consuming experimental evolution work, it is beneficial to have a tractable system such as the bobtail squid–*Vibrio* mutualism that is ideal for manipulation and co-evolution research.

## Conclusion

**Fig. 3.**
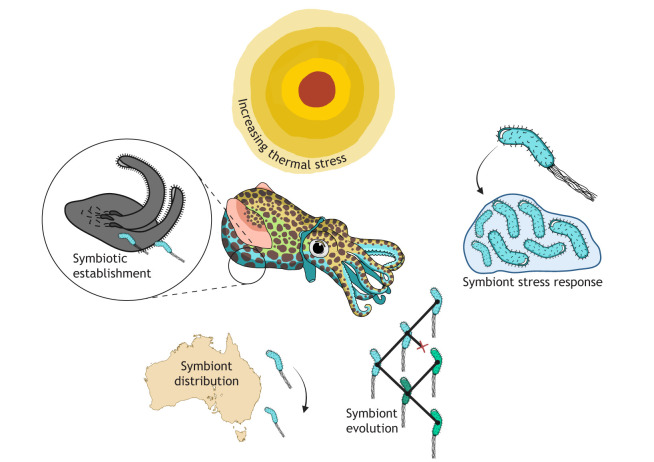
**The impacts of thermal stress on symbiotic cooperation and organismal fitness.** As thermal stress increases (top) it will impact many dimensions of organismal fitness, including the relationship between host organisms and their symbionts. The beneficial association between bobtail squid (center) and their *Vibrio fischeri* symbionts (right), residing in the squid's light organ (left), can provide valuable insights into multiple dimensions of these changing dynamics. Topics that can be interrogated include how increased thermal stress alters symbiont behavior (upper right) and decreases symbiotic fitness and establishment (left), whether thermal stress informs symbiont distribution (bottom left) and whether thermal stress imposes evolutionary pressures on microbes over shorter time scales than the long-term association with their squid host (bottom right). Created in BioRender by Thieme, M., 2026. https://BioRender.com/t80mnj8. This figure was sublicensed under CC-BY 4.0 terms.

Impacts of thermal stress on organismal fitness, from the perspective of symbiotic cooperation, are diverse and challenging to study (Fig. 3). The extensively researched sepiolid squid–*Vibrio* system has provided insight into microbial symbioses for over 30 years (McFall-Ngai and Montgomery, 1990; McFall-Ngai, 1999; Nyholm and McFall-Ngai, 2021). The complex biochemical communication between bobtail hosts and *Vibrio* has been studied at length studied, revealing key mechanisms susceptible to thermal stress that are highly conserved across eukaryotic–bacterial interactions (Brooks and Mandel, 2016; Otjacques et al., 2025a). The mutualism with bobtail squid has provided greater insight into the delicate balance between mutualism and parasitism for bacteria, which often teeters when disrupted by temperature (Nishiguchi and Nair, 2003; Patz et al., 2005; Ruby et al., 2005; Dunn, 2012). The ecology of the bobtail squid–*Vibrio* symbiosis has unveiled the impact of temperature on both symbiont environmental distribution and host preference (Nishiguchi, 2000; Jones et al., 2006). Co-evolution, a major theme in symbiosis biology, has been systematically studied in this relationship – even to the point where symbionts have been experimentally adapted to thermal stress and shown changes in host association proficiency (Cohen et al., 2019). These results, and the amenability of the bobtail squid–*Vibrio* association to manipulation, provide an ideal context in which to interrogate the range of mechanisms susceptible to environmental stressors, including changes in temperature (Bosch et al., 2019). The advancements and discoveries from the bobtail squid–*Vibrio* system provide a critical basis for new research approaches to understand how shifting thermal ranges are impacting not just organisms but microbe-dependent organismal systems.
